# Supplementary material for: Exploring the Impact of Climatic Variables on Arecanut Fruit Rot Epidemic by Understanding the Disease Dynamics in Relation to Space and Time
Source: J Fungi (Basel). 2022 Jul 19;8(7):745. doi: 10.3390/jof8070745 (PMC9319122; doi:10.3390/jof8070745)
Supplement: Supplementary file 1 [file jof-08-00745-s001.zip › jof-1805530-supplementary.pdf]

2018

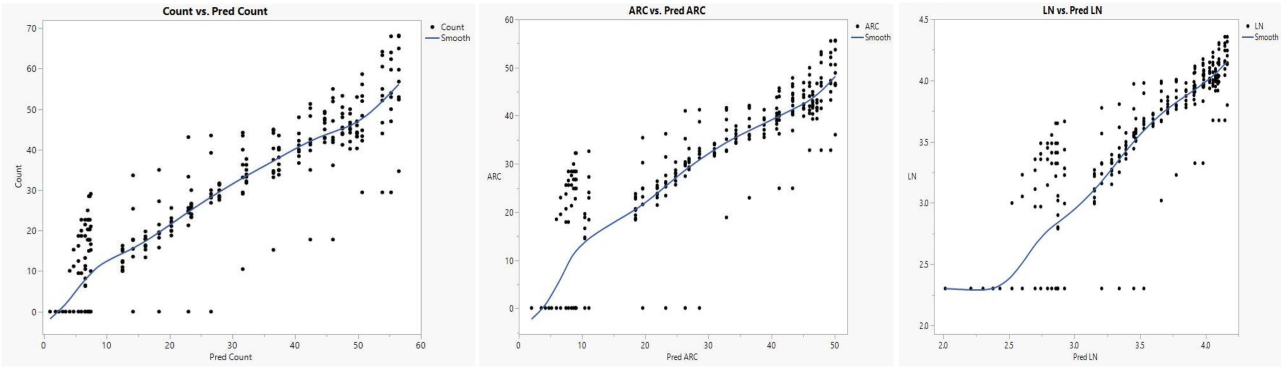

2019

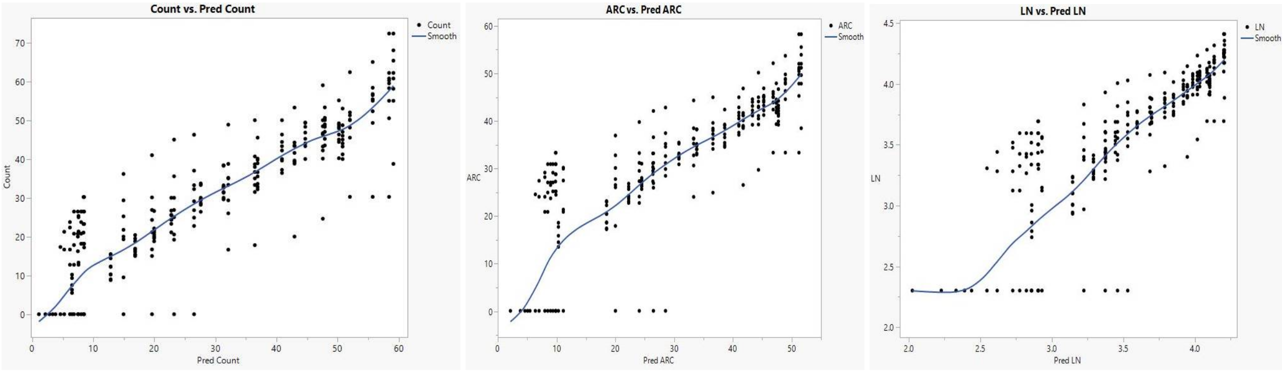

Supplementary Figure S1. Summary and estimates of generalized linear model (GLM) with negative binomial regression model with log link including different model parameters (count, ARC, and linear (LN)).

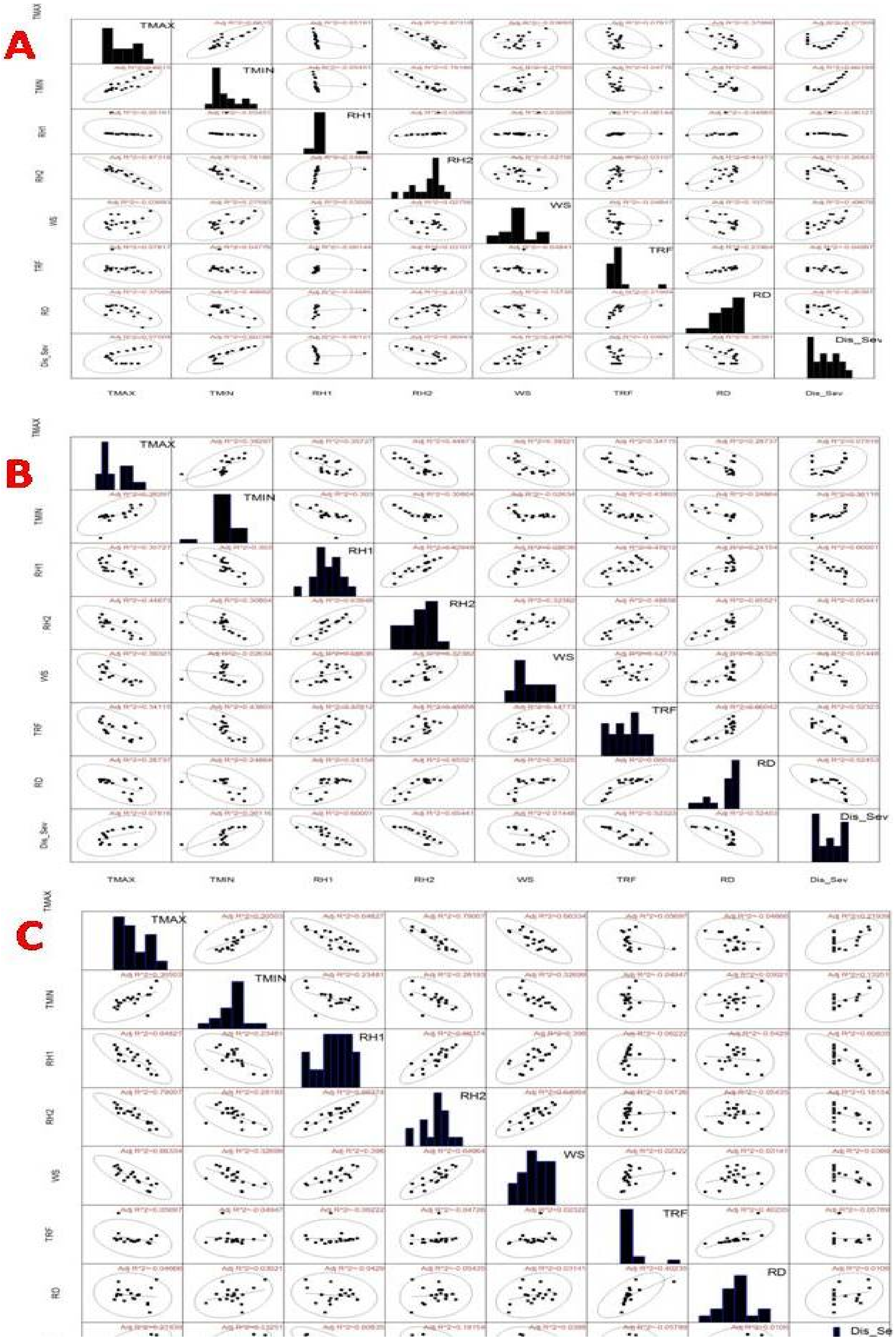

Supplementary Figure S2. Regression analysis on regional occurrence of FRD pitting climatic predictors in Malnad (**A**), Coastal (**B**), and Maidan (**C**) regions of Karnataka.
